# Advancing Our Understanding of Molecular Mechanisms in Radiation-Induced Normal Tissue Toxicity

**DOI:** 10.3390/genes17091157

**Published:** 2026-09-21

**Authors:** Rupak Pathak

**Affiliations:** Division of Radiation Health, Department of Pharmaceutical Sciences, College of Pharmacy, University of Arkansas for Medical Sciences, Little Rock, AR 72205, USA; rpathak@uams.edu

Radiation-induced normal tissue toxicity remains one of the most significant barriers to the safe and effective use of radiation in cancer therapy [1,2,3,4] and a major concern in radiation exposure scenarios [5,6,7]. Understanding the molecular events that govern tissue injury, repair, and long-term disease progression is essential for developing effective mitigation strategies [8]. The articles presented in this Special Issue provide a broad and multidisciplinary perspective on the molecular mechanisms underlying radiation-induced normal tissue toxicity, highlighting advances in proteomics, inflammation biology, vascular dysfunction, cellular senescence, regenerative medicine, and emerging therapeutic technologies.

A recurring theme throughout this issue is the application of systems-level approaches to identify molecular pathways that determine tissue responses to ionizing radiation. The review by Larrey and Pathak examined the use of proteomics to characterize radiation-induced gastrointestinal injury, emphasizing alterations in proteins involved in oxidative stress, inflammation, apoptosis, mitochondrial dysfunction, and tissue regeneration [9]. Such studies demonstrate the growing value of proteomic technologies for identifying biomarkers of radiation injury and uncovering novel therapeutic targets.

Expanding upon this systems biology theme, the article by Suman employed an integrative proteomic and bioinformatics approach to investigate radiation-induced senescence-associated secretory phenotype (SASP) factors in renal cortical epithelial cells and their potential role in renal carcinogenesis [10]. By analyzing the SASP Atlas, GENT2, Human Protein Atlas, and TCGA datasets, the study identified tissue-specific radiation-induced SASP proteins and revealed the dual nature of senescence-associated signaling [10]. Notably, ALDH18A1 and ASPH emerged as radiation-induced SASP factors associated with unfavorable renal cancer prognosis, while other SASP components displayed potentially protective functions [10]. These findings highlight the importance of cellular senescence and SASP-mediated microenvironmental changes in linking radiation exposure, chronic tissue injury, and long-term cancer risk. The work further illustrates how integrative proteomic analyses can uncover novel biomarkers and therapeutic targets for mitigating delayed radiation effects [10].

Several contributions in this issue emphasize the central role of inflammation and innate immune signaling in radiation-induced tissue damage. The review by Pawar and colleagues highlighted the involvement of Toll-like receptor 4 (TLR4) signaling in radiation-induced heart disease, demonstrating how persistent activation of inflammatory pathways contributes to oxidative stress, fibrosis, adverse cardiac remodeling, and cardiovascular dysfunction [11]. These findings identify innate immune pathways as promising targets for intervention in late radiation effects.

The importance of vascular biology in radiation injury is further underscored by the work of Binz and Pathak, who identified genomic mosaicism in human primary endothelial cells and demonstrated the presence of a terminal chromosome Xq deletion in endothelial cells [12]. Their findings remind investigators that genomic heterogeneity within experimental models can significantly influence endothelial responses to radiation and may affect interpretations of vascular injury and tissue repair mechanisms.

Translation of mechanistic discoveries into practical countermeasures is exemplified by the study from Kumar and colleagues, which demonstrated that placenta-derived stromal cells (PLX-R18) enhance hematopoietic recovery and improve survival following total-body irradiation [13]. By modulating inflammatory responses and stimulating regenerative pathways, PLX-R18 represents a promising medical countermeasure against radiation-induced bone marrow injury and illustrates the therapeutic potential of cell-based interventions [13].

The Special Issue also broadens the discussion to genetic susceptibility and metabolic regulation. The work by Fujiwara’s group examined disease associations involving drug-metabolizing enzymes, highlighting pathways that may influence individual susceptibility to environmental stressors, including radiation [14]. Such research contributes to the growing framework of precision radiation medicine, where genetic and molecular profiles may one day guide individualized risk prediction and therapeutic decision-making.

Finally, emerging technological innovations are represented by the review on nanoparticle-based approaches in radiation oncology. Nanotechnology offers opportunities to improve tumor targeting, enhance radiosensitization, optimize drug delivery, and reduce collateral damage to healthy tissues as shown by Girigoswami’s group [15]. These advances have the potential to improve the therapeutic outcome and minimize normal tissue toxicity during radiation treatment.

Collectively, the articles in this Special Issue demonstrate that radiation-induced normal tissue toxicity is governed by a complex interplay among oxidative stress, inflammation, innate immune signaling, vascular dysfunction, cellular senescence, metabolic regulation, and tissue regeneration. The integration of proteomics, molecular biology, genomics, and translational therapeutics provides unprecedented insight into these interconnected pathways. We hope that the findings presented in this issue stimulate further interdisciplinary collaboration and accelerate the development of biomarkers, medical countermeasures, and targeted therapies that protect normal tissues while preserving the indispensable benefits of radiation in medicine and public health.

## Data Availability

No new data were created or analyzed in this study. Data sharing is not applicable to this article.
